# A Study on the 3D Deformation Behavior of Porous PDMS Flexible Electronic Composite Films Stretched under Different Temperatures

**DOI:** 10.3390/ma16196586

**Published:** 2023-10-06

**Authors:** Cheng Chen, Ziyun Li, Yanlai Wang, Ze Zhang, Chunhua Ren

**Affiliations:** School of Mechanical Engineering, Tianjin University of Commerce, Tianjin 300134, China; 120220284@stu.tjcu.edu.cn (Z.L.); wyl17695598404@163.com (Y.W.); 120210272@stu.tjcu.edu.cn (Z.Z.);

**Keywords:** porous PDMS flexible electronic composite films, digital image correlation, strain fluctuation, off-plane displacement

## Abstract

Flexible electronic films need to be applied in different ambient temperatures. The porous substrate of the composite film enhances air permeability. The lifespan of these composite films is significantly affected by variations in temperature and substrate porosity. To explore the impact of temperature and porosity on the performance of composite films, we developed a 3D deformation detection system utilizing the advanced three-dimensional digital image correlation (3D-DIC) method. This system enabled us to observe and analyze the 3D deformation behaviors of porous polydimethylsiloxane (PDMS) flexible composite films when they are subjected to uniaxial stretching at different temperatures. We proposed employing two parameters, namely the strain fluctuation coefficient (*M*) and off-plane displacement (*w*), to characterize the 3D deformation of the films. This holistic characterization of deformation through the combined utilization of parameters M and *w* held greater significance for composite films compared to the conventional practice of solely measuring mechanical properties like the elastic modulus. Through experimental analysis, we discovered that as the temperature increased, the *M* value of the film decreased while the *w* value increased for the same stretching distance. Furthermore, the porosity of the composite film depended on the doping mass ratio of PDMS to deionized water during the fabrication process. Specifically, when the ratio was set at 6:1, the composite film exhibited the smallest *M* value and *w* value, and the highest air permeability. Additionally, the 3D deformation behavior remained stable across different temperatures for this specific ratio. Moreover, our findings unveiled a remarkable association between the parameter *w* and the resistance value of the device. These findings provide valuable insights for optimizing the fabrication process of porous PDMS flexible electronic composite films.

## 1. Introduction

Flexible electronic composite films hold immense promise in applications such as health monitoring, smart wearable devices, and flexible displays [1,2,3]. However, these films face the challenge of withstanding diverse forms of deformation under varying ambient temperatures, which puts their mechanical properties to the test [4,5].

A flexible electronic composite film consists of a composite material featuring a metal interconnect layer and a flexible substrate structure [6]. The metal interconnect layer, known for its high elastic modulus and mechanical strength, effectively absorbs most mechanical deformations in practical scenarios [7]. Extensive research conducted by multiple teams has led to the design of various wire structures for the metal interconnect layer—serpentine, linear, horseshoe, and wave, to name a few [8,9,10]. Through numerous mechanical performance tests, it has been determined that the two-dimensional horseshoe-shaped metal interconnect structure exhibits superior mechanical capabilities [11]. 

Thin film patterning is a vital stage in the production of flexible electronic composite films and, currently, photolithography represents the predominant technique for achieving this. In recent years, however, several research groups have explored the utilization of laser direct writing technology to create metal patterns on polymer films [12,13]. This innovative approach eliminates the requirement for masks and presents advantages such as shorter process cycles, enhanced material utilization rates, and reduced environmental pollution. Nevertheless, it is important to note that the heat generated during laser irradiation affects the substrate, thereby demanding the use of substrate materials with exceptional temperature resistance in this method.

Among flexible substrates, polymers are commonly used, and polydimethylsiloxane (PDMS) stands out due to its excellent ductility [14]. To improve the air permeability of the composite film, it is common practice to create a porous structure within the substrate. However, due to the high hydrophobic nature of PDMS, conventional porogenic agents like deionized water are unable to penetrate the interior of PDMS. As a result, the desirable polymer characteristics of the porous PDMS substrate are preserved [15]. In addition to deionized water, various substances such as carbon nanoparticles, sugar particles, and oxygen have been identified as potential porogenic agents for the creation of porous PDMS [16,17,18]. This approach enhances the air permeability of the composite film. Studies have revealed that the level of porosity significantly impacts the mechanical properties of porous structural composite films [19]. 

In challenging application environments, flexible electronic composite films must endure not only mechanical deformations like tensile, bending, and torsion but also function reliably across varying temperatures [20,21,22]. Exceeding the film’s deformation threshold in three dimensions can lead to failure and subsequent loss of functionality, resulting in substantial economic losses. Therefore, understanding the 3D deformation behavior of flexible electronic composite films in different application environments and optimizing their mechanical properties accordingly remain areas ripe for exploration.

Over the years, numerous scholars have conducted a range of mechanical property tests on flexible electronic composite films, including tensile, bending, compression, and similar assessments [23,24,25]. Typically, the mechanical properties of these composite films are evaluated based on criteria such as modulus of elasticity and tensile strength at break. However, these criteria exhibit limitations when applied to the complex deformation behavior of composite films. While some research teams employ finite element simulation to determine the stress–strain distribution under various conditions [26,27], accurately modeling the intricate adhesion between the metal interconnect layer and flexible substrate remains a challenge. Only a few studies have ventured into detecting the global 3D deformation patterns of flexible electronic composite films in different environments using a system that assesses mechanical properties through multivariate parametric metrics. 

Among the available inspection methods, non-contact inspection offers the highest accuracy in capturing the working condition of films [28,29]. Three-dimensional digital image correlation (3D-DIC), a non-contact detection method leveraging binocular vision, calculates strain and displacement fields during the deformation process by analyzing grayscale information from images captured before and after deformation. This method boasts advantages such as high measurement sensitivity and real-time full-field measurement. As a result, it has found widespread application in deformation tests for composite membranes and is well-suited for measuring the overall 3D deformation of flexible electronic composite films [30,31,32]. 

This study delved into the 3D deformation characteristics of porous PDMS flexible electronic composite films subjected to different temperature conditions using the 3D-DIC method. The analysis primarily focused on examining how temperature changes influence the extent of tensile deformation in these composite films. To comprehensively evaluate the mechanical properties, two parameter indicators—the strain fluctuation coefficient (*M*) and off-plane displacement (*w*)—were employed. Additionally, this study proposed an optimal doping mass ratio of PDMS to deionized water for fabricating porous PDMS substrates, providing valuable insights for optimizing the properties of flexible electronic composite films.

## 2. Materials and Methods

### 2.1. Porous PDMS Flexible Electronic Composite Film Preparation Process and Equipment

To prepare the porous PDMS flexible electronic composite film, deionized water was used as the porogenic agent, resulting in substrates with varying porosities by modifying the mass ratio of PDMS and deionized water doping. After conducting preliminary experiments, we observed that the mass ratio of PDMS to deionized water significantly influences the flexibility and transparency of the porous substrate. Adhering to the standards of flexibility and transparency for flexible electronic composite films, four groups of substrates were created with different mass ratios (PDMS/deionized water): 2:1 (Group A), 4:1 (Group B), 6:1 (Group C), and 8:1 (Group D). Additionally, a control group consisting of pure PDMS substrate was established (Group E).

**Figure 1:**
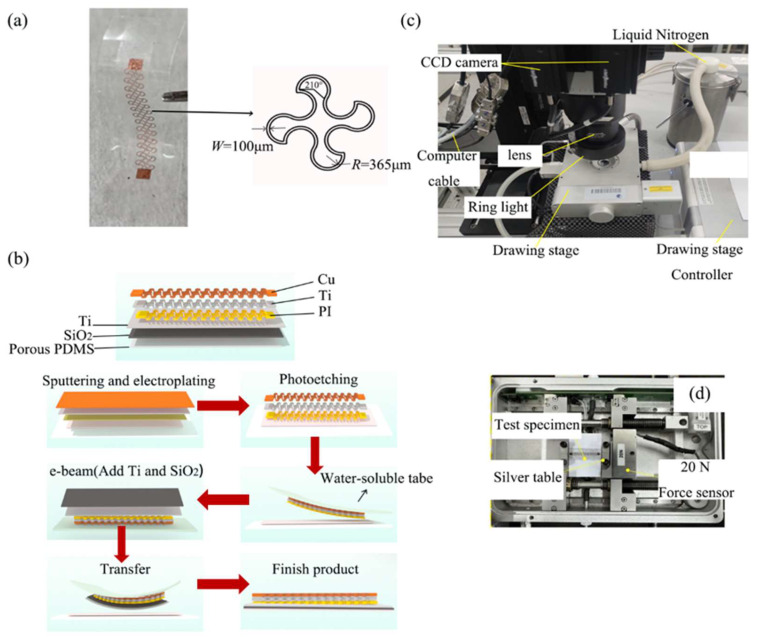
(**a**) Photograph of porous PDMS flexible electronic composite film. (**b**) Fabrication process for porous PDMS flexible electronic composite film. (**c**) Three-dimensional deformation detection system for flexible electronic composite film. (**d**) Picture of composite film mounted on stretching table.

Figure 1a showcases a photograph of a sample flexible electronic composite film fabricated using porous PDMS. This composite film comprises a porous PDMS substrate, a bonding layer made of Ti and SiO_2_, and a metal interconnect layer composed of Cu, Ti, and polyimide (PI). The fabrication process consists of several crucial steps, encompassing substrate preparation, sputtering, electroplating, photoetching, and transfer [33], as shown below.

Step 1. Porous PDMS Prepolymer Preparation: Mechanical stirring of PDMS and deionized water with different mass ratios yielded the porous PDMS prepolymer. The prepolymer was then spin-coated on a high-quality float glass plate and baked in a vacuum oven at 120 °C for 40 min, resulting in a 300 μm-thick porous PDMS film.

Step 2. Metal Interconnect Layer and Bonding Layer Preparation: Pure PDMS was spin-coated on a float glass plate, followed by baking in a vacuum oven at 80 °C for 40 min to form a film. Subsequently, a layer of polyimide (PI) was spin-coated on the PDMS after ultraviolet ozone (UVO) treatment. The PI was further baked at 250 °C for 90 min. Next, a 5 nm-thick Ti layer and a 100 nm-thick Cu layer were sputtered onto the PI, followed by electroplating a 2 μm-thick Cu film at 2 V. Photolithography, development, and etching techniques were employed to fabricate the two-dimensional horseshoe-shaped metal interconnect layer.

Step 3. Transfer Printing: The metal interconnect layer was removed using water-soluble tape. Then, a 5 nm-thick Ti layer and a 50 nm-thick SiO_2_ layer were applied through electron beam evaporation (e-beam). After UVO treatment, the interconnection layer was hot-pressed with the previously prepared porous PDMS film, resulting in the fabrication of the porous PDMS flexible electronic composite film, as illustrated in Figure 1b.

To facilitate deformation inspection, a 3D DIC measurement system was constructed, comprising 3D DIC software (Vic-3D v7, Correlated Solutions, Irmo, SC, USA), a miniature precision tensile stage (Linkam TST350, Manufactured by Linkam, UK), two POINT GREY industrial cameras (GS3-PGE-50S5M-C models, each with a resolution of 2448 pixels by 2048 pixels), an OLYMPUS lens (SDF-PLAPO-1XPF model), and a ring halogen light source (with a power control range of 0 to 50 W), as depicted in Figure 1c.

### 2.2. The 3D-DIC Method Principle and Film Permeability Testing

The 3D-DIC method employed in this study utilizes pre-prepared scattering spots, known as feature points, on the device under test to capture displacements. By tracking the gray values of these feature points in images, the method can accurately determine the displacements. Based on the principle of binocular stereo-vision imaging, the method utilizes two industrial cameras that simultaneously record images of the same scene from different orientations. This allows for the identification of corresponding points present in both images. Leveraging the pre-calibrated internal and external parameters of the cameras, the method calculates the three-dimensional coordinates of the points in a human-defined coordinate system in space [34,35]. 

**Figure 2:**
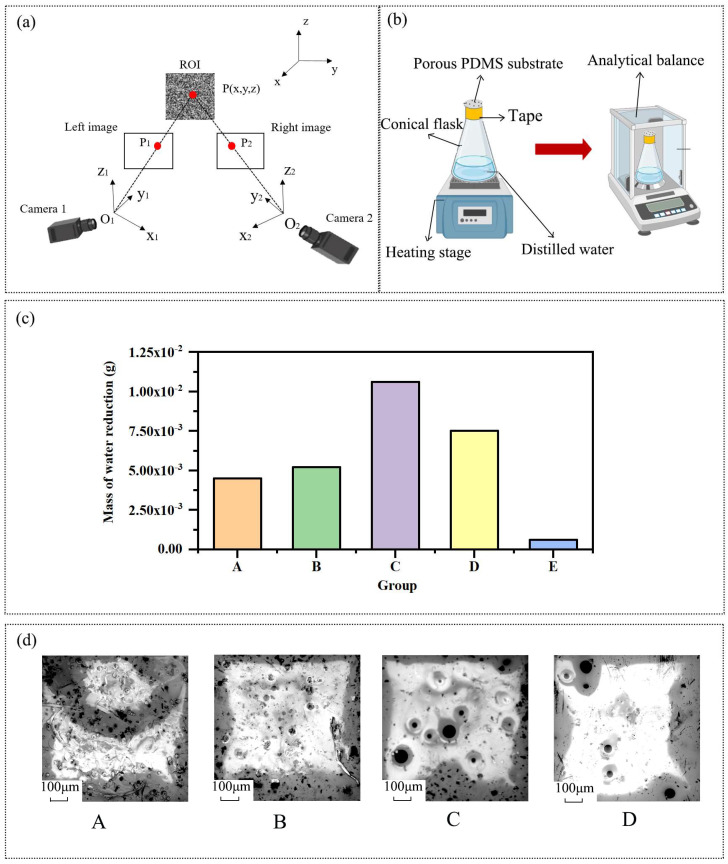
(**a**) Principle of binocular stereo vision. (**b**) System for testing the air permeability of films. (**c**) Mass of distilled water reduced by boiling in five sets of conical flasks. (**d**) SEM images of Groups A–D.

A simplified schematic illustrating the principle of binocular stereo vision is presented in Figure 2a. The optical centers of the left and right industrial cameras are denoted as O_1_ and O_2_, respectively. The point P to be measured within the region of interest (ROI) is imaged at point P_1_ in the image plane of camera 1 and point P_2_ in the image plane of camera 2. Through binocular stereo vision, the specific coordinates of point P in real space (the intersection of line O_1_P_1_ and line O_2_P_2_) can be determined from the coordinates of P_1_ and P_2_. However, if only one camera is used, the specific location of point P cannot be ascertained.

To analyze the deformation characteristics, the ROI is divided into subzones, with the distance between the subzones adjusted by setting the step size. The 3D-DIC system then determines the coordinates of the feature points in all subzones based on individual point coordinate determination. Initially, the system captures the coordinates and grayscale information of subzones before deformation. Then, using a subpixel search algorithm [36], it tracks the subzones after deformation. To match the subzones before and after deformation, the system utilizes the cross-correlation function [37]. Subsequently, it calculates the displacements of all subareas in all directions after deformation, thereby obtaining the 3D deformation features of the entire ROI.

To evaluate the permeability of various porous PDMS substrates, a dedicated film permeability testing system was designed and constructed as shown in Figure 2b. Each of the five groups of substrates was placed at the opening of a conical flask containing 10.0 g of distilled water and securely wrapped with high-temperature resistant tape. These flasks were then subjected to boiling on a heated stage for a duration of 15 min. Following the boiling process, the conical flasks from each group were removed and weighed. The reduction in mass of distilled water before and after boiling was calculated to quantify the amount of water vapor that passed through the different substrate groups during the boiling process. This allowed for a comparison of the permeability among substrates with varying doping ratios.

For every group of substrates, three independent experiments were conducted, and the average of the three experiments was determined as the final mass reduction of the distilled water. The results, illustrating the mass reduction of distilled water in the conical flasks for each group, are presented in Figure 2c. Notably, the porous PDMS substrate demonstrated significantly improved permeability compared to the pure PDMS substrate. Among the different groups of porous PDMS substrates, Group C had the highest amount of water vapor permeating through them in a given time period which exhibited the highest permeability.

To validate these findings, we acquired surface images of four different sets of porous substrates using a scanning electron microscope (Phenom XL 10020-L, Manufactured by Phenom, The Netherlands) at a magnification of 260×. Amongst the obtained images, we carefully selected representative ones (displayed in Figure 2d) that effectively demonstrate the porosity of the substrates. Notably, the images highlight that Group C substrates exhibited the largest pore aperture and the most concentrated pore distribution.

Despite the higher specific gravity of deionized water in Group A and Group B substrates compared to Group C substrates, both A and B substrates exhibited a considerable presence of air bubbles. Notably, Group A displayed a significantly higher concentration of air bubbles than Group B. However, these air bubbles were all confined and did not contribute to enhancing the permeability of the substrates.

If the specific gravity of deionized water surpassed a certain threshold, extensive water droplets enclosed by the PDMS tended to form within the substrate after stirring and spin-coating with PDMS. These water droplets subsequently underwent a transformation into sealed bubbles rather than permeable pores during the drying process through baking. Consequently, there exists an optimal doping ratio of deionized water during the preparation of porous substrates, with Group C substrates closely aligning with this ratio.

### 2.3. Experimental Methods 

The hot and cold tensile table’s temperature was adjusted incrementally to 0 °C, 10 °C, 20 °C, 30 °C, 40 °C, and 50 °C using a temperature sensor placed within the table. Liquid nitrogen was utilized for cooling, while the resistance wire within the silver table was used for heating. The five groups of specimens with scattering spots were sequentially mounted on the tensile table for tensile experiments at each of the six temperatures (0 °C, 10 °C, 20 °C, 30 °C, 40 °C, and 50 °C), as shown in Figure 1d. The brightness of the annular halogen light source was optimized for clear imaging. To obtain highly accurate deformation patterns, images were captured at every 100 μm strain within a strain range of 0–5 mm. In total, 50 sets of images were taken for each group of specimens stretched at a specific temperature level.

The precision of the measurement system was evaluated through a zero-deformation experiment before the tensile test. To minimize experimental inaccuracies, three repetitive experiments were conducted for each group of specimens. Subsequently, the 3D-DIC software imported 50 sets of images, and the region of interest was defined as the six horseshoe-shaped joints located at the center of the metal bonding layer. Utilizing a step size of 7 pixels and a subregion size of 39 pixels by 39 pixels, deformation results were obtained for groups A–E at six temperatures (0 °C, 10 °C, 20 °C, 30 °C, 40 °C, and 50 °C) for tensile strains ranging from 0 to 5 mm. 

We performed cyclic tensile tests on the five sets of specimens at a temperature of 20 °C to investigate the three-dimensional deformation behavior under cyclic loading. The cyclic stretching range was set as between 0 and 5 mm. Through preliminary experiments, it was determined that the wire would fracture after approximately 300 cycles. Hence, we conducted tests for 50, 100, 150, 200, and 250 cycles, each with a cyclic stretching speed of 1 mm/s.

To further evaluate the behavior, we employed a DC low-resistance tester (RK2511BL) with a measuring range of 0.1 mΩ–50.0 kΩ. This allowed us to assess the resistance values of the five sets of samples subjected to different degrees of stretching (ranging from 0 to 5 mm) at six distinct temperatures: 0 °C, 10 °C, 20 °C, 30 °C, 40 °C, and 50 °C.

## 3. Results

**Figure 3:**
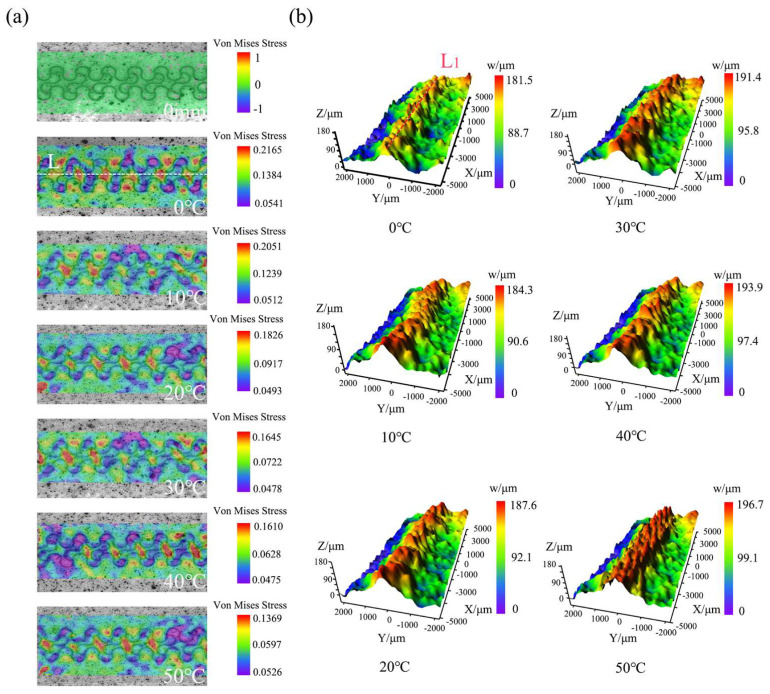
(**a**) Von Mises strain field of Group C devices stretched by 3 mm at 0 °C, 10 °C, 20 °C, 30 °C, 40 °C, and 50 °C. (**b**) Three−dimensional off−plane displacement field of Group C devices stretched by 3 mm at 0 °C, 10 °C, 20 °C, 30 °C, 40 °C, and 50 °C.

The effect of temperature on the 3D deformation behavior of porous PDMS flexible electronic composite films was thoroughly examined by observing the 3D deformation process of each device group stretched at various temperatures using the advanced 3D-DIC system. The Von Mises strain field (Figure 3a) and 3D off-plane displacement field (Figure 3b) of group C devices were meticulously analyzed during their 3 mm stretch, conducted at five distinct temperatures.

As indicated in Figure 3a, the Von Mises strain values of the porous PDMS flexible electronic composite film displayed an interval distribution when subjected to stretching. The larger strain values gathered in the red region located at the center of the two-dimensional horseshoe shape. This region represents the strain value of the substrate, which lacks a metal layer distribution. Conversely, the purple region with smaller strain values appears at the four edges of the horseshoe shape. These edges have a dense distribution of the metal interconnection layer, indicating the strain value of this layer. This phenomenon occurs due to the substantial difference in elastic modulus between the porous PDMS substrate and the metal interconnect layer. Consequently, during the stretching process, a strain mismatch arises between the two layers, leading to what we term the strain mismatch of the flexible electronic composite film made of porous PDMS.

This strain mismatch phenomenon has a significant impact on the adhesion between the metal interconnect layer and the porous PDMS substrate. If the adhesion is not strong enough, it can result in delamination and warpage of the metal interconnect layer, especially when the phenomenon becomes more intense. To investigate the influence of temperature change on strain mismatch, we carefully analyzed the entire strain field. We observed a decreasing trend in the strain difference between the maximum and minimum values within the region of interest as the temperature increased from 0 °C to 50 °C for the Group C devices while undergoing the same 3 mm stretch. Hence, it can be concluded that as the temperature increases, the degree of strain difference between the metal interconnect and the porous PDMS substrate decreases, subsequently weakening the strain mismatch phenomenon.

In Figure 3b, the porous PDMS flexible electronic composite film exhibits an overall off-plane displacement under stretching, characterized by a large center region and a small edge region. This behavior arises due to the tendency of the metal interconnection layer to separate from the porous PDMS substrate during stretching. As a result, a significant off-plane displacement occurred in the center region of the device where the metal layer was dense, while a small off-plane displacement was observed in the edge region where there was no metal layer. For the Group C specimens stretched for 3 mm from 0 °C to 50 °C, the maximum value of off-plane displacement in the region with a dense metal layer distribution increased from 181.5 μm to 197.6 μm. This indicates that the separation tendency of the metal interconnect layer from the substrate intensifies with increasing temperature.

To accurately assess the 3D deformation intensity of the devices, horizontal lines *L* and *L*_1_ were drawn in the regions of interest of the Von Mises strain field and the 3D off-plane displacement field, respectively. The region where line *L* passes through exhibited the most significant strain value change, indicating a probable strain mismatch phenomenon throughout the region of interest. Similarly, the area where line *L*_1_ intersects represents the most significant off-plane deformation of the metal interconnection layer.

**Figure 4:**
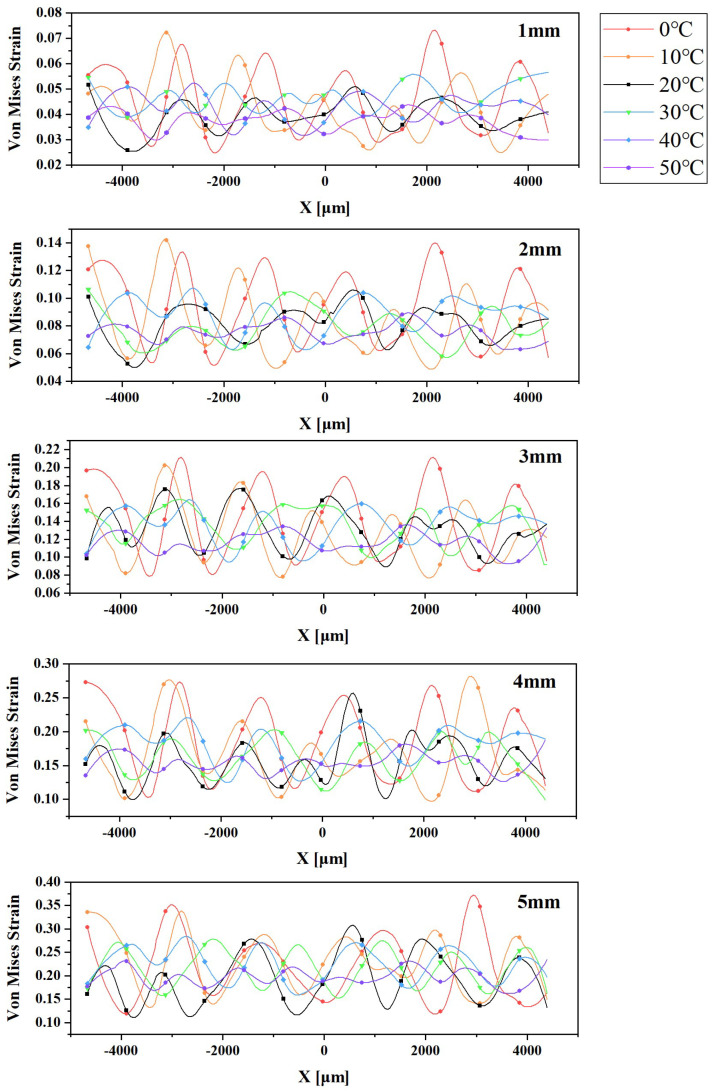
Von Mises strain curve on line *L* at 0 °C, 10 °C, 20 °C, 30 °C, 40 °C, and 50 °C when the Group C devices are stretched by 1−5 mm.

To further analyze the strain mismatch, we extracted the strain value data of all pixel points on line *L* and plotted the curve shown in Figure 4, using *L* as the horizontal coordinate. The fluctuation amplitude of the curve effectively characterizes the degree of strain mismatch for the Von Mises strain. The results demonstrate that the fluctuation amplitude of the Von Mises strain values decreases with increasing temperature for each tensile distance between 1 mm and 5 mm, indicating a weakening degree of strain mismatch. These findings align with our previous preliminary analysis.

To precisely quantify this degree of strain mismatch, we introduced the strain fluctuation index *M* [38]. This index represents the standard deviation of all strain values on line *L*, which effectively avoids the impact of sudden strain changes at certain locations resulting from other interfering factors. Consequently, it provides more reasonable characterizations. The strain fluctuation index *M* is calculated using the following formula:

(1) $$M=\sqrt{\frac{1}{s-1}\sum_{\left(x,y\right)}^{s}{\left[\varepsilon \left(x,y\right)-\bar{\varepsilon }\right]}^{2}}$$

**Figure 5:**
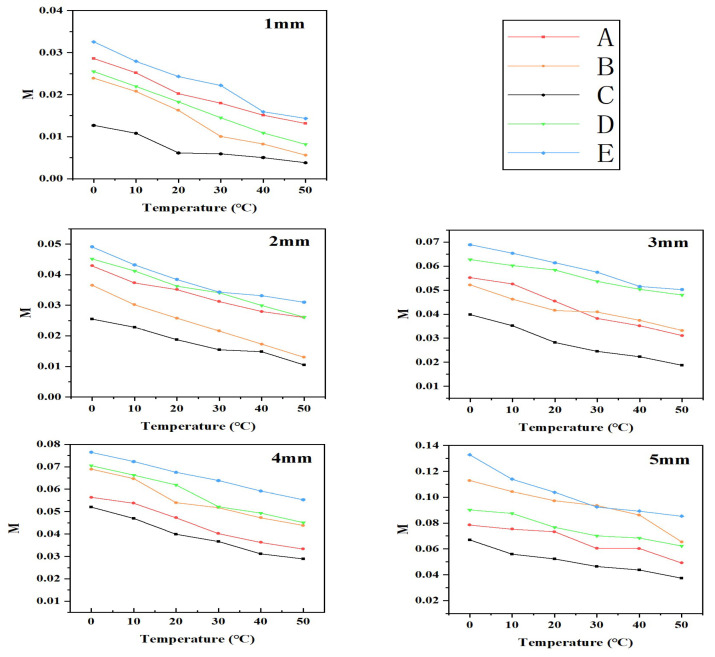
Line graph of strain fluctuation index *M* for five groups of devices A–E stretched 1–5 mm at 0–50 °C.

In the above equation, (x,y) denotes the pixel coordinates within the calculation area, ε(x,y) represents the strain value at that particular pixel coordinate, $\bar{\varepsilon }$ is the average strain value, and *s* represents the total number of pixels in the calculation area. The value of *M* reflects the degree of strain mismatch between the metal interconnect layer and the porous PDMS substrate. *M* was computed for devices A–E, stretched from 1 mm to 5 mm at temperatures ranging from 0 °C to 50 °C. The results are presented in Figure 5.

Figure 5 illustrates that the strain fluctuation index *M* generally decreases as the temperature increases for each tensile distance within the range of 1 mm to 5 mm. This phenomenon can be attributed to the rise in ambient temperature, which promotes molecular motion within the porous PDMS substrate. As a result, the elastic modulus of the substrate increases, thus reducing the difference between the elastic moduli of the substrate and the metal layer. Consequently, the degree of strain mismatch between them decreases, leading to a decline in the strain fluctuation index *M*.

Furthermore, we extracted the off-plane displacements (*w*) of all pixel points on line *L*_1_ from the 3D off-plane displacement field and calculated the average value (*w_m_*) to quantify the degree of off-plane deformation in the metal interconnect layer of the device. The average value (*w_m_*) was calculated using the following formula:

(2) wm=∑i=1nwin

**Figure 6:**
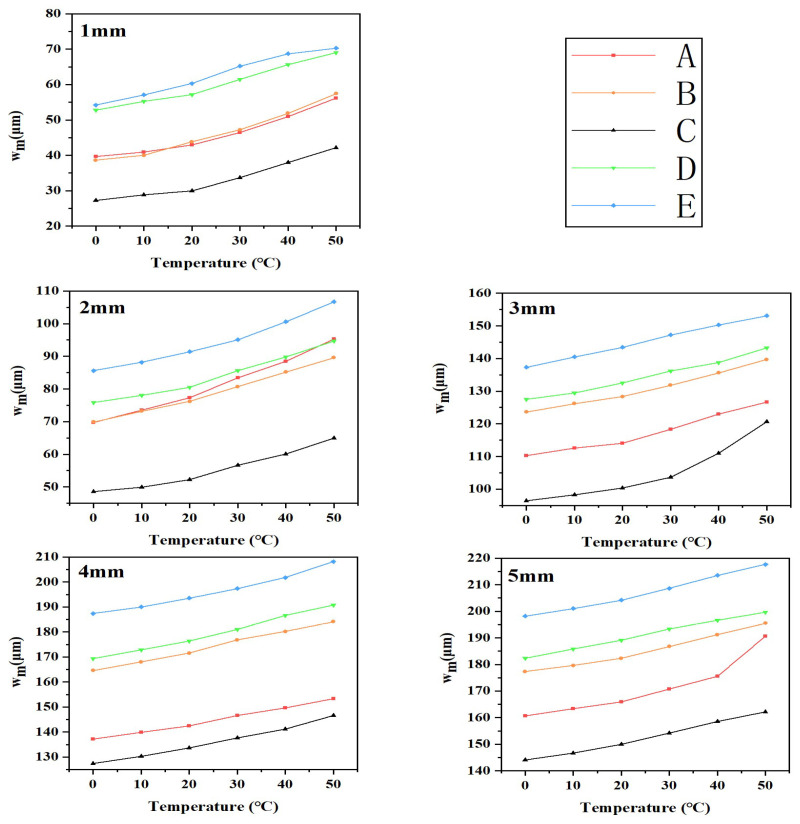
Line graph of the average off-surface displacement *w_m_* of line *L*_1_ for five groups of devices A–E when stretched 1–5 mm at 0–50 °C.

In the above equation, *w_i_* represents the off-plane displacement of all pixel points in the region where line *L*_1_ passes through, while *n* represents the total number of pixel points within that region. *w_m_* was calculated for the five groups of devices at temperatures ranging from 0 °C to 50 °C, stretched from 0 mm to 5 mm. The results are displayed in Figure 6.

Figure 6 demonstrates that as the temperature increases, the off-plane displacements of each specimen group steadily increase. This behavior can be attributed to the thermal expansion of the porous substrate, leading to increased off-plane displacement between the metal interconnecting wires and the porous polydimethylsiloxane (PDMS) substrates.

**Figure 7:**
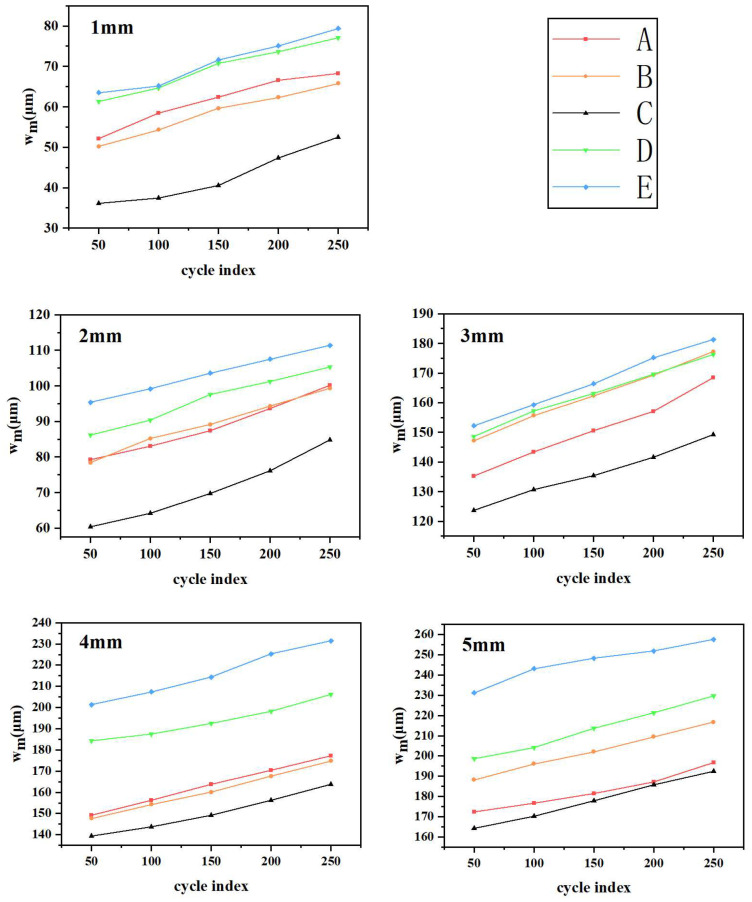
Graphs of the average off-plane displacement (*w_m_*) during cyclic stretching (0–5 mm) at 20 °C for five groups of devices labeled A–E.

Moreover, during the cyclic tensile tests, all five sets of samples demonstrated distinct levels of displacement from their respective surfaces. To quantify this behavior, we calculated the average off-plane displacement of the specimens in each set when subjected to stretching between 0 and 5 mm for a total of five cycles at a temperature of 20 °C. These results are graphically presented in Figure 7.

Figure 7 portrays the observed trend of off-plane displacements for the devices undergoing cyclic stretching compared to those subjected to a single stretch. Notably, a clear pattern emerges: as the number of stretch cycles increased, the average off-plane displacement of all five device groups generally amplified. Remarkably, none of the five device groups experienced fracture even after enduring up to 250 cycles of cyclic stretching. This outcome underscores their exceptional resistance to fatigue.

**Figure 8:**
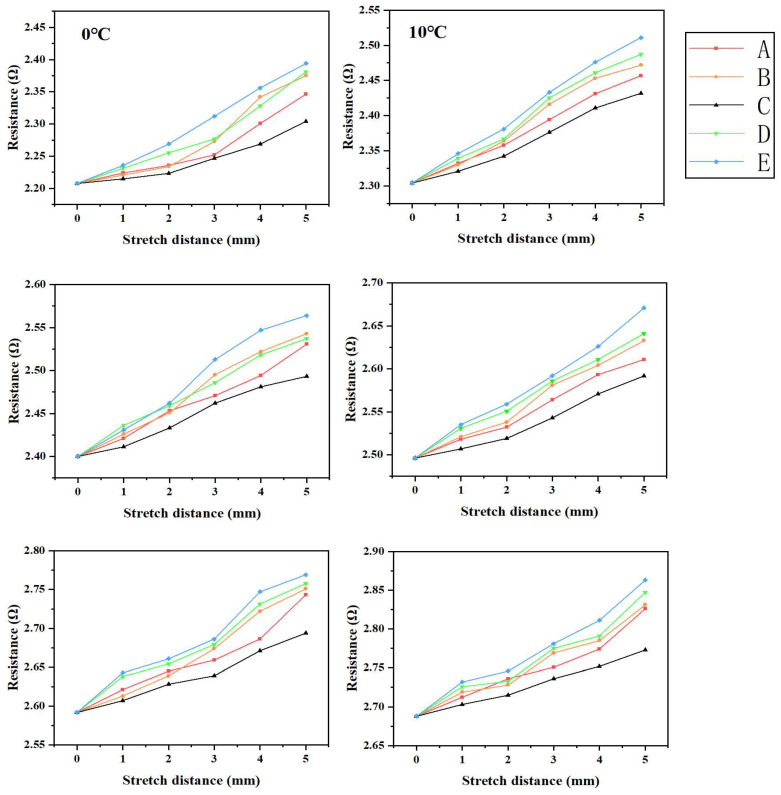
The resistance values of five device groups (A–E) when stretched between 1 and 5 mm at temperatures ranging from 0 to 50 °C.

Figure 8 presents the measured resistance values of wires stretched from 0 to 5 mm across five different groups of specimens at six varying temperatures (0 °C, 10 °C, 20 °C, 30 °C, 40 °C, and 50 °C). These results provide valuable insights into the behavior of the specimens under different temperature conditions.

In accordance with Figure 8, the device consistently exhibited remarkable electrical conductivity despite variations in temperature and stretching distance. This can be attributed to the inherent high electrical conductivity of copper. Additionally, the resistance value of the device demonstrated a positive correlation with both temperature and stretching distance, indicating its adaptability to different environmental conditions.

An intriguing discovery emerged during our research: at identical temperatures and stretching distances, devices that exhibited greater off-surface displacement also displayed higher resistance values. This phenomenon can be attributed to the elongation of the two-dimensional horseshoe-shaped wires in all directions caused by the off-plane displacement generated through device stretching. Consequently, the overall lengthening of the wires contributes to an increase in their resistance value.

The findings indicated that when compared to devices with pure PDMS substrates, devices with porous PDMS substrates exhibited lower values for both the strain fluctuation index *M* and the average off-surface displacement *w_m_* at all temperatures and tensile distances. The mean off-surface displacements of *w_m_* during different cycles of stretching were consistently low. This suggests that the porous substrate not only enhances the permeability of the composite film but also improves its mechanical properties. This improvement arises from the structure of the porous PDMS substrate, which helps alleviate residual stresses when the metal interconnect layer and the flexible substrate undergo inconsistent deformation under loads. Furthermore, different porosity levels result in varying performance, with Group C devices (with a mass ratio of 6:1) generally exhibiting lower *M* and *w_m_* values compared to the other groups. This observed regularity suggests that devices in this group possess the most stable mechanical properties under different ambient temperatures. Proper doping of deionized water effectively enhances device performance. It is noteworthy that the formation of excessive closed bubbles within the substrate leads to a reduction in both permeability and mechanical properties, rather than an improvement. Thus, the optimal doping ratio for Group C’s devices has been identified in this study.

In summary, these findings provide valuable insights into the influence of temperature on the 3D deformation behavior of porous PDMS flexible electronic composite films. They highlight the importance of considering strain mismatch and off-plane displacement in the design and application of such films, ultimately contributing to the development of more reliable and robust devices.

## 4. Conclusions

In conclusion, we successfully fabricated porous PDMS flexible electronic composite films by incorporating deionized water. This innovative approach not only enhances the air permeability of the films but also significantly improves their mechanical properties compared to using pure PDMS substrates. To investigate the 3D deformation behavior of these composite films under different temperature conditions, we employed a state-of-the-art 3D-DIC-based system for accurate measurement and analysis. Our study focused on four sets of porous PDMS flexible electronic composite films with varying porosities. By carefully analyzing the Von Mises strain field and the 3D off-plane displacement field of the composite films under different environmental temperatures, we gained valuable insights into their deformation characteristics. In order to quantify and assess the 3D deformations, we introduced two key parameters: the strain fluctuation coefficient *M* and the off-plane displacement *w*. In addition, cyclic stretching experiments were conducted to ascertain the off-plane displacements of different devices with varying porosities and numbers of cyclic stretching. Subsequently, resistance values of these devices were measured at different temperatures, unveiling a relationship between the wire resistance values and off-plane displacements.

After analyzing the experimental phenomena, we obtained the following conclusions:

(1) As the temperature increased, the strain fluctuation coefficient *M* in the porous PDMS flexible electronic composite films decreased. This implied a weaker strain mismatch phenomenon occurring at the same stretching distance. Furthermore, we observed an increase in the off-plane displacement *w* value as the temperature increased, indicating a higher off-plane deformation of the metal interconnecting layer within the composite films.

(2) The optimum mechanical properties and air permeability were achieved with a mass ratio of 6:1 between PDMS and deionized water. This composition yielded the most stable and desirable performance for the composed films.

(3) Porous PDMS flexible electronic composite films exhibited remarkable fatigue resistance, showcasing their exceptional durability. With an increase in the number of cyclic stretching cycles, there was a corresponding increase in the off-plane displacement of the device.

(4) Porous PDMS flexible electronic composite films displayed exceptional electrical conductivity in various environmental conditions. Moreover, there existed a direct correlation between the off-plane displacement of the device and its resistance, leading to higher resistance values as the displacement increases.

Through this comprehensive investigation, we have uncovered important insights into the deformation behavior of porous PDMS flexible electronic composite films when subjected to various temperature environments. These findings provide valuable insights for optimizing the fabrication process of porous PDMS flexible electronic composite films. By carefully controlling the doping mass ratio and understanding the influence of temperature on the film’s properties, we can enhance their flexibility and temperature stability, thus extending their lifespan and expanding their potential applications.

## Data Availability

Data are contained within the article.
